# A Cardiovascular Conundrum: A Case of Excessive Exercise Masquerading as a Heart Attack

**DOI:** 10.7759/cureus.46407

**Published:** 2023-10-03

**Authors:** Piyush Puri, Ridhi Bhagat, Deepak Singla, Kamaljot Kaur Ahuja, Hari P Pokala

**Affiliations:** 1 Internal Medicine, Adesh Institute of Medical Science and Research, Bathinda, IND; 2 Internal Medicine, Teerthanker Mahaveer Medical College and Research Centre, Moradabad, IND; 3 Internal Medicine, Government Medical College, Patiala, Patiala, IND; 4 Internal Medicine, Shri Guru Ram Rai Institute of Medical & Health Sciences, Patiala, IND; 5 Gastroenterology, HCA Northwest Hospital, Houston, USA

**Keywords:** cardiac troponin, troponin, mi, cardiology, exercise

## Abstract

Cardiac troponins T and I are sensitive biomarkers often associated with acute coronary syndrome but can also be elevated after intense exercise, posing diagnostic challenges. We present the case of a 42-year-old male cyclist who complained of chest pain during exercise with elevated troponin levels. A comprehensive evaluation ruled out cardiac pathology but revealed acid reflux. Excessive cycling posture exacerbates reflux, likely contributing to chest pain and troponin elevation. This case underscores the importance of considering alternative etiologies in athletes with chest pain and elevated troponin levels after extreme exertion. It also highlights the role of antireflux therapy and activity modification in managing such cases. Further research is needed to elucidate the long-term cardiac effects of exercise-induced troponin elevation, although this is currently considered a reversible physiological phenomenon.

## Introduction

Troponin T and I are cardiac troponins released from myocardial cells following cardiac injury and can be acute or chronic [1]. Although highly suggestive of acute coronary syndrome, such as myocardial infarction, cardiac troponins can also be raised due to acute cardiac stress following strenuous exercise, as seen in athletes [2]. An increase in troponin levels following endurance exercise usually returns to baseline within 24 hours and does not warrant any treatment [3]. Exercises such as cycling and weightlifting also lead to increased intra-abdominal pressure, causing impaired motility of the esophagus, precipitating acid reflux, and acute exacerbation of gastroesophageal reflux disease (GERD) in patients with pre-existing GERD [4] which can be managed with appropriate pharmacotherapy [5].

## Case presentation

**Figure 1:**
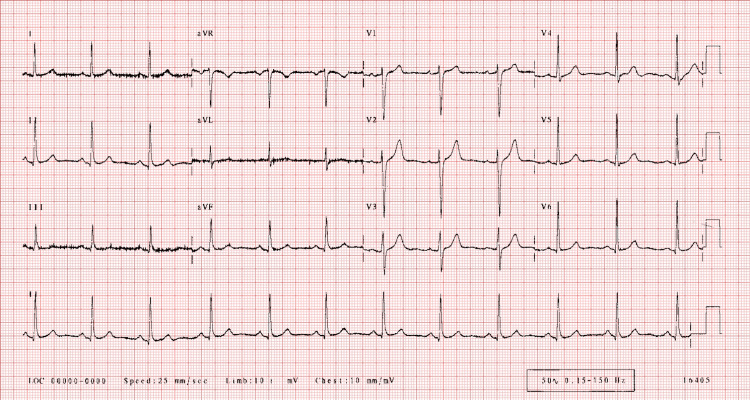
EKG findings. Normal sinus rhythm with heart rate in the 70-80s. Normal axis and no abnormal axis deviation. No left ventricular hypertrophy. No significant ST/T abnormalities are present.

**Figure 2:**
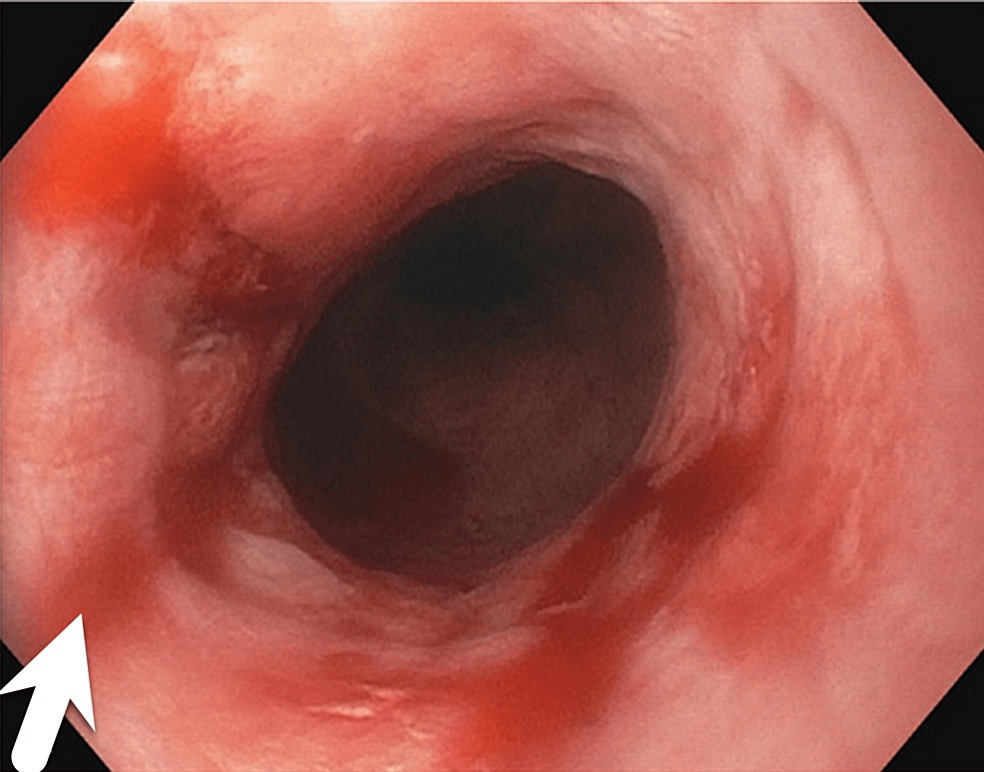
Endoscopy showing features of acid reflux. Arrow shows the esophagitis from the acid reflux.

A 42-year-old man presented to the emergency room (ER) with chest pain during cycling. His vitals on presentation were a heart rate of 102 beats/minute, SpO_2_ of 96%, and a blood pressure of 144/84 mmHg, and he was afebrile. He had no relevant medical history and was not taking any prescription medication. He was a non-smoker and non-alcoholic, with a body mass index of 20 kg/m^2^. The patient’s family history was negative for any cardiac events. EKG showed normal sinus rhythm (Figure 1). Echocardiography was normal and showed normal wall motility, ejection fraction of 55-60%, and normal valvular structure. No wall motion abnormalities were seen on echocardiography. Labs were sent, and the troponin I and brain natriuretic peptide (BNP) levels were 0.060 mg/mL and 63 pg/mL, respectively. The patient was given the option to undergo coronary angiography, but as he arrived in the ER with a normal EKG, ondansetron and pantoprazole were administered. The patient began feeling better. He was advised to undergo coronary angiography but the patient refused to undergo any invasive procedure as he had started to feel better and was in a healthy shape. He also refused to undergo CT angiography to avoid radiation exposure. Troponin I was assessed every three hours and trended as follows: 0.060 ng/mL > 1.20 ng/mL > 0.088 ng/mL > 0.02 ng/mL > 0.00 ng/mL. He was medically treated with aspirin, clopidogrel, and atorvastatin. The chest pain subsided a few minutes into the ER. Upon inquiring, the patient said he was a fitness enthusiast and would usually bicycle 80 miles every other day. He reported that he sometimes had acid reflux at night, three to four times a week. He would take over-the-counter omeprazole for the acid reflux. The patient had never experienced symptoms such as rapid heartbeat, shortness of breath, trembling, sweating, nausea, lightheadedness, numbness, chills or hot flashes, feelings of detachment, and fear of dying. Hence, panic attacks and anxiety were ruled out by history-taking when the patient arrived in the ER. Gastroenterology was consulted, and after reviewing the patient, an endoscopy was performed, which revealed features of acid reflux in the lower esophagus (Figure 2). The next day, the patient came for a follow-up, and an exercise stress test was done. The exercise stress test was insignificant.

Due to the curved posture during excessive bicycling, he would have increased acid reflux due to increased intra-abdominal pressure. It has been postulated that excessive exercise leads to acid reflux and chest pain. Exercise resulted in acute myocardial injury, which caused an increase in troponin levels.

He was advised to stop excessive exercise and was prescribed pantoprazole for a month with a follow-up in the clinic. The patient reported an improvement in his acid reflux symptoms and no chest pain ever since.

Differential diagnoses for these symptoms can be pulmonary embolism, esophageal spasm, peptic ulcer disease, panic attacks or anxiety, asthma or exercise-induced bronchoconstriction, costochondritis, etc. Diagnosing these potential conditions involves tailored approaches: myocardial infarction needs EKG and cardiac enzymes; GERD relies on reflux history and endoscopy; musculoskeletal causes require examinations and imaging; other cardiac issues may need stress tests or imaging; lifestyle factors are assessed based on habits; pulmonary causes involve imaging or lung function tests; gastrointestinal problems are diagnosed via endoscopy or ultrasound; psychological causes require psychological evaluation; respiratory issues use pulmonary function tests; costochondritis is confirmed clinically; referred pain relies on history and examinations; vascular issues may need CT or MRI; and medication-related causes involve reviewing medication history. It is a customized diagnostic process, leveraging various assessments to pinpoint the specific condition accurately.

## Discussion

Cardiac troponins are myocardial regulatory proteins that regulate the calcium-mediated action of actin on myosin. Cardiac forms of troponins (T and I) are encoded by specific genes and may theoretically be distinct from each other [1]. Cardiac troponin controls the contraction of cardiomyocytes. If cardiac troponin rises or falls above the upper limit, myocardial damage is diagnosed. Therefore, cardiac troponin measurement is part of a routine workup if acute coronary syndrome is suspected [2]. What is interesting, however, is that exercise often causes an increase in serum cardiac troponin, which peaks approximately three hours post-exercise and returns to basal levels within the next 24 hours. A growing body of evidence indicates that exercise-induced cardiac troponin elevation occurs in seemingly healthy athletes and may be a physiological acute reaction to exercise rather than a pathological symptom [6]. The addition of high-sensitivity troponin tests (unmatched in sensitivity for low myocardial damage) has made it possible to better characterize exercise-induced cardiac troponin elevation. This has resulted in the observation that measurable cardiac troponin changes are common not only with high levels of exercise (e.g., marathon) but also after normal exercise or a treadmill test (e.g., treadmill run). The source of this release of biomarkers and whether it represents a physiological or pathological pathway remain controversial [7]. Troponin release may be associated with cardiac adaptation to exercise, in which transient myocardial (myocardial) injury serves as a signal for adaptation, resulting in improved structural and functional function. In acute cases, ultra-endurance exertion may cause a decrease in global left ventricular (diastolic and systolic), and in the vast majority of cases, this is accompanied by an increase in circulating serum markers of cardiac damage, such as troponin (T and I), myoglobin (myoglobin + creatine kinase), creatine kinase (creatine kinase-myocardial band), and BNP [8]. Cardiac troponin T detection after cycling races was also found to be about 50% lower than that of runners after cycling events. The increase in cardiac troponin T was also found to increase with the duration of an endurance event and the body weight of the participant [9]. The changes in body position required during competitive cycling or weightlifting reduce the benefits of gravity on esophageal clearance as the body moves from an upright posture. The increase in intra-abdominal pressure caused by the “Valsalva” maneuver performed by the weightlifter or the “Bentover Racing” position maintained by the cyclist both increases the abdominal portion of the vesicle force that pushes gastric contents upward against the bottom of the esophagus [4].

In addition to palpitations, chest pain, etc., athletes also suffer from upper gastrointestinal symptoms such as heartburn, belching, epigastric pain, regurgitation, nausea, and vomiting. These symptoms have been reported in athletes in a variety of sports, including running, cross-country skiing, rowing, weightlifting, and cycling [4]. While runners appear to have more total gastrointestinal symptoms, the proportion reporting only upper symptoms is 30% and the proportion reporting only lower symptoms is 60% [5]. Although exercise is beneficial in the long term, acute bouts of exercise increase the risk of adverse events (including sudden cardiac death). The most common adverse reaction after long periods of strenuous activity is an increase in cardiac troponin. This is observed in almost all runners who finish a marathon, and more than half of them exceed the upper limit. Cardiac troponin above the upper limit indicates myocardial damage and can cause clinical confusion in otherwise healthy athletes [2]. Consequently, the mechanisms that lead to post-exercise elevation in cardiac troponins remain a matter of debate. Elevations in cardiac troponin were initially thought to be irreversible because the heart was thought to be a post-mitotic organ with cardiomyocytes that could not be regenerated or replaced. Therefore, the release of cardiac troponin is thought to be pathognomonic of necrosis [3]. Post-exercise increases in cardiac troponin have also been observed in adolescent athletes. However, the differences in cardiac troponin I levels in adolescents and adults are not consistent. It has previously been suggested that elevated exercise-induced cardiac troponin in younger athletes may be due to the immaturity of the myocardium in adolescents compared to adults. Based on this hypothesis, younger athletes may respond to exercise with elevated levels of this biomarker [6]. Exercise may also result in altered gastrointestinal motor activity, such as a decrease in the activity of the esophageal peristaltic, a decrease in the tone of the esophageal sphincters, and delayed emptying of gastric contents. These and other physiological changes may occur secondary to disruption in the mesenteric circulation during exercise [4]. Exertional gastroesophageal reflux (GER) is most common in athletes with GER at rest but may occur only during exercise. Symptoms tend to worsen with increasing exercise intensity, longer exercise sessions, and a fed versus fasting state [4]. Treatment should be continued for at least eight weeks. At this time, individuals need to decide if they want to continue treatment for an indefinite amount of time, or if they want to start a step-down approach. The goal of the step-down approach is to determine the minimum amount of medication required for symptom relief [5].

## Conclusions

The most likely explanation is that the patient experienced exercise-induced troponin elevation along with chest wall pain secondary to gastric reflux exacerbated by his cycling position. This case reinforces the need to consider alternative etiologies when athletes present with chest pain and elevated biomarkers after extreme exertion. It also underscores the importance of comprehensive cardiac testing to exclude dangerous conditions.

Further research is required to better understand the long-term cardiac effects, if any, of repeated bouts of pronounced troponin release following excessive endurance exercise. However, at present, transient exercise-induced troponin elevations are thought to represent a reversible physiological phenomenon without lasting sequelae. This case supports conservative management with activity cessation and anti-reflux therapy in athletes who develop chest pain and elevated troponins after intense exertion but have no other evidence of irreversible myocardial injury.
